# Analysis of newly established EST databases reveals similarities between heart regeneration in newt and fish

**DOI:** 10.1186/1471-2164-11-4

**Published:** 2010-01-04

**Authors:** Thilo Borchardt, Mario Looso, Marc Bruckskotten, Patrick Weis, Julia Kruse, Thomas Braun

**Affiliations:** 1 Max-Planck-Institute for Heart and Lung Research, Parkstr. 1, 61231 Bad Nauheim, Germany

## Abstract

**Background:**

The newt *Notophthalmus viridescens *possesses the remarkable ability to respond to cardiac damage by formation of new myocardial tissue. Surprisingly little is known about changes in gene activities that occur during the course of regeneration. To begin to decipher the molecular processes, that underlie restoration of functional cardiac tissue, we generated an EST database from regenerating newt hearts and compared the transcriptional profile of selected candidates with genes deregulated during zebrafish heart regeneration.

**Results:**

A cDNA library of 100,000 cDNA clones was generated from newt hearts 14 days after ventricular injury. Sequencing of 11520 cDNA clones resulted in 2894 assembled contigs. BLAST searches revealed 1695 sequences with potential homology to sequences from the NCBI database. BLAST searches to TrEMBL and Swiss-Prot databases assigned 1116 proteins to Gene Ontology terms. We also identified a relatively large set of 174 ORFs, which are likely to be unique for urodele amphibians. Expression analysis of newt-zebrafish homologues confirmed the deregulation of selected genes during heart regeneration. Sequences, BLAST results and GO annotations were visualized in a relational web based database followed by grouping of identified proteins into clusters of GO Terms. Comparison of data from regenerating zebrafish hearts identified biological processes, which were uniformly overrepresented during cardiac regeneration in newt and zebrafish.

**Conclusion:**

We concluded that heart regeneration in newts and zebrafish led to the activation of similar sets of genes, which suggests that heart regeneration in both species might follow similar principles. The design of the newly established newt EST database allows identification of molecular pathways important for heart regeneration.

## Background

Some urodele amphibians possess an extraordinary capacity for regeneration [1,2]. The newt *Notophthalmus viridescens *(order caudata, family Salamandridae, genus Notophthalmus) can completely regenerate limbs, tail, jaws, lens and retina after amputation or following attacks of natural predators. Interestingly, newts are also able to regenerate internal organs such as parts of the central nervous system [3] and parts of the ventricles of the heart after amputation or mechanical damage [4-6] suggesting that they exhibit a general program that enables regenerative responses. This ability distinguishes newts (and also teleost fish) from other vertebrates, which have lost (or supress) the capacity for comprehensive organ regeneration. Mammals, for example, are not able to repair substantial cardiac injuries by replacement with functional tissue. Instead, the mammalian heart responds with scar formation and fibrosis resulting in severe functional impairment (reviewed in [7,8]). The failure of mammalian hearts to regenerate seems to be caused by the inability of cardiac myocytes to proliferate and a preference for hemostasis and fibrosis, which occurred during evolution, together with the advent of high-pressure circulation [9,10].

Newt cardiomyocytes are virtually non-proliferative in undamaged hearts but respond to cardiac injuries by proliferation [4,5,11,12]. At present, it is not clear whether proliferating cardiomyocytes arise solely from resting cardiomyocytes, which might undergo partial de-differentiation or re-programming, or also from dedicated cardiac stem cells that are able to replace lost cardiac tissue. Despite this open question it is undisputed that newt cardiomyocytes re-enter the cell cycle in inductive environments. The plasticity of newt cardiomyocytes to adopt a different cellular fate is also demonstrated by their ability to contribute to skeletal muscle formation in regenerating limbs [13].

Understanding gene expression during critical stages of cardiac regeneration is a fundamental prerequisite to decode the molecular processes, which enable newt cardiomyocytes to re-enter the cell cycle and functionally replace, lost cardiac tissue. Considering its exceptional regenerative capabilities, surprising little sequence information is available for the newt. By the end of 2008, 125 entries were listed in the NCBI database, which encode a maximum of 83 non-redundant proteins including 5 very short sequences. Another 12 unique sequences were present in the nucleotide database resulting in a total number of around 100 unique sequences, which correspond to expressed genes from *N. viridescens*. Limited EST datasets exist so far for two other urodele amphibians; the Mexican axolotl (*Ambystoma mexicanum*) and the Eastern tiger salamander (*A. tigrinum tigrinum*) [14,15]. Both species belong to the family of Ambystomatidae (mole salamander) that have separated from the Salamandridae family after divergence of family-level Salamander lineages more than 150 million years ago [16,17]. The paucity of annotated sequence information for newts severely compromises efforts to analyze changes in the transcriptional profile during regeneration. This is emphasized by a recent attempt to characterize transcriptional changes during nerve dependent limb regeneration. Due to the low degree of sequence annotation, the combination of microarray and 454 sequencing analysis yielded only a limited number of new contigs after assembly of 454 cDNA sequences with existing expressed sequence tags (ESTs) even though the number of non-redundant human-*A. mexicanum *orthologous sequences was increased considerably [18].

The size of the newt genome, archived on 22 chromosomes is estimated to be around 10 times larger than most mammalian genomes [19,20] which impedes attempts to determine the complete genomic sequence. We therefore decided to concentrate on expressed sequences. This reduces the complexity of sequence information that needs to be analyzed but allows a comprehensive view on genes expressed during cardiac regeneration in the newt. In this study, we focused on newt hearts 14 days after damage, the time when highest cell proliferation during heart regeneration occurs [4,11]. We have assembled and annotated a large set of sequence data resulting in the identification of a group of potential open reading frames, which seem to be unique for urodele amphibians. Furthermore, a web based relational database was constructed that combines sequence data and functional annotations. The database was used to compare transcriptional signatures of damaged hearts of newts and zebrafish, which is the only other known vertebrate capable of cardiac regeneration.

## Results and Discussion

### Analysis of EST quality and contig assembly

**Figure 1:**
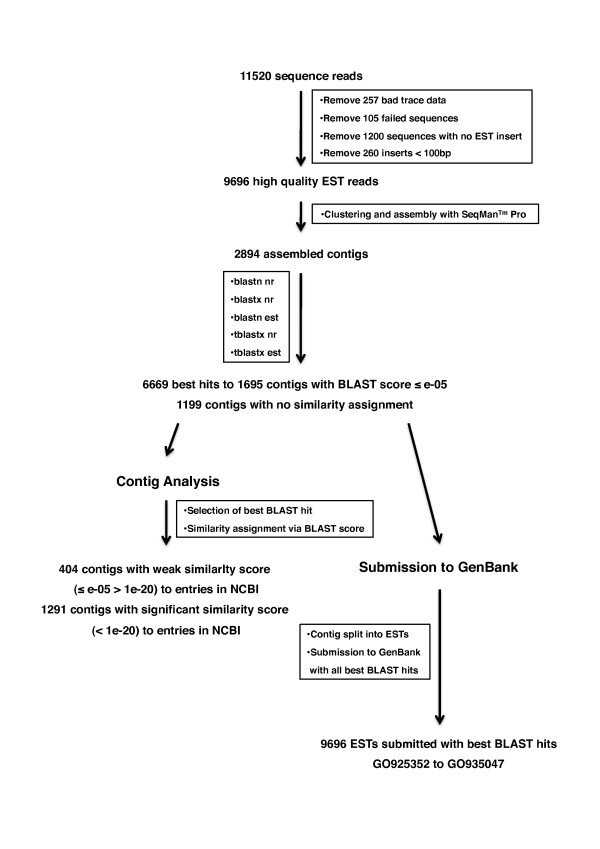
**Flowchart of EST sequence processing for contig analysis and GenBank submission**. 11520 sequence reads were subtracted from bad trace data, failed sequence reads, empty vectors, and short transcripts to obtain 9696 high quality ESTs. Assembly of ESTs into 2894 contigs was followed by BLAST searches with 5 different algorithms to obtain 6669 best hits for 1695 contigs with scores ≤ 1e-05. Hits with smallest score were selected for contig analysis to evaluate weak and significant similarities to NCBI entries and to evaluate the distribution of organisms with closest similarity. Contigs were split into their respective ESTs for submission to GenBank. Best hits from all BLAST algorithms were attached to facilitate further analysis.

To obtain a comprehensive non-biased view of gene activity in regenerating newt hearts we constructed a non-normalized cDNA library from damaged hearts 14 days after mechanical injury. 100,000 randomly selected clones were picked and transferred into 384 well plates. We selected 11520 clones for 5'end sequencing, which resulted in 11158 high quality reads. After clipping of flanking vector sequences and long polyA tails with a custom made software tool, 1200 sequences which did not contain any cDNA insert were removed. Furthermore, 260 ESTs were discarded since they contained sequences shorter than 100 bp in length, thus yielding 9696 EST sequences of high quality (Figure 1). The majority of ESTs were in the range of 500-700 bp with a median size of 537 bp. 137 EST reads (4.7%) had a length of more than 1000 bp (Figure S1 in additional file 1). The quality of our sequence data with respect to read lengths and contig sizes was similar to previous EST sequencing projects focusing on *A. mexicanum *[14].

We assembled the 9696 ESTs into 2894 contigs with the commercial software seqman™ Pro using default parameters. The average contig length was determined to be 627 bp. Most contigs were in the range of 900 bp (Figure S2a) in additional file 1). Since the newt heart library was not normalized, we quantified the EST distribution per contig to determine the redundancy level of our EST dataset. 2000 ESTs (69.1%) were only present as singleton sequences, whilst 387 contigs (13.3%) contained only two EST clones, a ratio that is very similar to the EST sequencing projects of *A. mexicanum *[14,15]. On average, the number of ESTs per contig was 3.1, which confirmed the high diversity of our dataset. 130 contigs, assembled from 3949 sequence reads, contained more than 20 ESTs (Figure S2b in additional file 1), most probably representing transcripts with highest abundance at this stage of heart regeneration.

### BLAST searches, homology assignment, and EST annotation

To identify putative homologies to known nucleotide and protein sequences, we performed separate BLASTn, BLASTx and tBLASTx searches for the 2894 assembled contigs in NCBI nr nucleotide and EST databases. 1695 contigs with a BLAST score of at least = 1e-05 were used for further analyses. According to the BLAST scores 404 contigs were categorized as weakly similar (≤ e-05 > 1e-20) and the remaining 1291 contigs in significantly similar (< 1e-20) to existing entries in the NCBI database (Figure 1). To further analyze which species showed the highest similarities for the individual contigs, we extracted BLAST hits with the smallest e-values from the different BLAST runs (Table S1 in additional file 2). Not surprisingly, 66.66% of all best hits were assigned to amphibians with the majority belonging to 15 identified salamander species, followed by 15.93% of best hits for 19 different mammalian species and 10.86% of best hits for 5 different avian species (Table S1 in additional file 2). Most of the best hits corresponded to EST sequences, which lack functional annotations or protein identities. However, BLASTx searches revealed significant similarities for many of these contigs to known protein entries in NCBI. Thus, for submission to GenBank all initial 2894 contigs were split into their respective ESTs and deposited together with the information about the best hits (score ≤ 1e-05) from all BLAST algorithms applied (Figure 1). All 9696 EST sequences were published in GenBank on July 30, 2009 under the accession numbers GO925352 to GO935047

### Analysis of contig sequences

To identify contig sequences encoding full open reading frames we screened all blastx searches with a score ≤ 1e-05 for similarities that start within the first 10 amino acids of their putative protein homologues. 273 out of 534 contigs, which showed significant similarities before amino acid position 10, also contained a polyA tail. 184 contigs contained both the start codon and a 3'polyA sequence suggesting that the complete open reading frame was covered (Table 1).

**Table 1:** Full-length contig analysis of BLAST hits with an E-value < 1-05.

alignment start amino acid position	number of contigs	contigs total	number of contigs full length (polyA)	Contigs full length total
1	384	384	184	184

2	32	416	20	204

3	24	440	16	220

4	28	468	16	236

5	18	486	8	244

6	10	496	7	251

7	16	512	9	260

8	9	521	5	265

9	13	534	8	273

543 contigs, which show an alignment to their putative homologues before amino acid position 10. 184 contigs were found to encode a full-length cDNA based on the presence of a putative initiation codon and a polyA tail.

**Figure 2:**
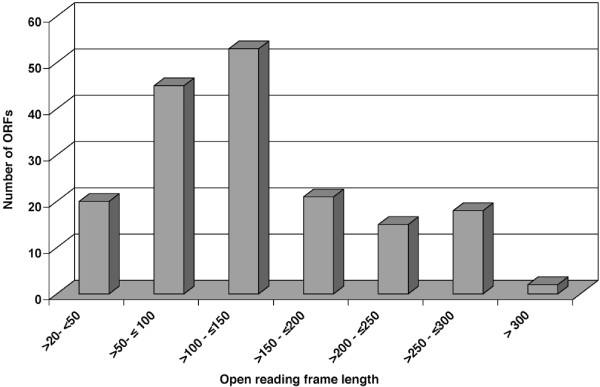
**Determination of potential open reading frames from BLAST hits with no identifiable homology**. 174 potential ORFs longer than 22 amino acids in length were identified by EST scan. 32% of all ORFs were longer than 150 amino acids.

No assignable similarity to existing data base entries was obtained for 1199 contigs (Figure 1). This ratio of 41.4% was slightly higher compared to the *A. mexicanum *EST sequencing project of Habermann et al. [14] (34% non-assignable sequences), which might be due to the smaller number of NCBI database entries for *N. viridescens *compared to NCBI entries for Ambystoma species. Many contigs, which lacked an allocated BLAST hit to existing NCBI database entries, mapped to 3'untranslated regions, as determined with the software ESTscan [21]. However, a substantial proportion of these contigs (32%) encoded open reading frames longer than 150 amino acids. In total, 174 contigs with ORFs longer than 22 amino acids were identified (Figure 2). We reason that these contigs might represent proteins that are unique to urodele amphibians or correspond to proteins, which to date have not yet been identified in related organisms due to incomplete genomic information. However, we cannot exclude that the identified ORFs represent parts of known proteins whose similarities are located in parts of the sequence that are not covered by the analyzed ORFs.

The number of EST clones which map to a certain contig will roughly reflect the relative abundance of corresponding mRNAs since the cDNA library from regenerating newt heart used in this study was not normalized. We found that 40.7% of all ESTs were contained in 38 contigs, indicating that this group represented the most abundantly expressed genes in regenerating newt hearts 14 days after damage. In fact, the contigs with the largest number of EST hits comprising 21.6% of all ESTs, represented members of the globin family. The second largest group of ESTs (7.8%) was derived from mitochondrial transcripts. Members of the actin-myosin cytoskeleton represented 2.8% of all sequenced inserts. Another 1.3% of all ESTs were classified as ribosomal proteins (Table 2). The large number of EST clones encoding globin mRNAs was most likely caused by an incomplete removal of nucleated blood cells from regenerating hearts.

**Table 2:** Gene families with highest abundance in the newt heart EST library.

Number of ESTs	Number of contigs	Identity	Abundance (%) total	Contig identity
2019	4	Globin family	21,60%	beta-globin, minor adult alpha-globin, Hemoglobin beta-2 subunit, major adult alpha-globin chain

755	6	Mitochondrial coded	7,80%	Paramesotriton hongkongensis mitochondrion, Notophthalmus viridescens voucher UTA 56597 cytochrome b (cytb) gene, partial cds; mitochondrial, Notophthalmus viridescens NADH dehydrogenase subunit I (ND1) gene, partial cds; tRNA-Ile, tRNA-Gln, and tRNA-Met genes, complete sequence; NADH dehydrogenase subunit II (ND2) gene, complete cds; tRNA-Trp tRNA-Ala, tRNA-Asn, tRNA-Cys, and tRNA-Tyr genes, complete sequence; and cytochrome c oxidase subunit I (COI) gene, partial cds; mitochondrial genes for mitochondrial products

271	7	Actin-Myosin Cytoskeleton	2,80%	X. laevis mRNA for myosin light chain, Cynops ensicauda b-actin mRNA for beta actin, Ambystoma mexicanum tropomyosin mRNA, Sus scrofa MyHC-slow mRNA for myosin heavy chain slow isoform, Xenopus laevis myosin light chain 1av (MLC1av) mRNA, HSTNCS Human mRNA for slow skeletal troponin C (TnC), Bufo marinus cardiac troponin I (Tnni3) mRNA

132	6	Ribosomal proteins	1,30%	Xenopus laevis similar to ribosomal protein S20, mRNA (cDNA clone MGC:52591 IMAGE:5542932), complete cds; Ribosomal protein L23 [Mus musculus] gb|AAH10114.1; Ictalurus punctatus ribosomal protein L36 mRNA, complete cds; Xenopus tropicalis 40S ribosomal protein S6, mRNA (cDNA clone MGC:76307 IMAGE:5379757), complete cds;Latimeria chalumnae ribosomal protein L7 mRNA, partial cds; Xenopus tropicalis 60S ribosomal protein L35, mRNA (cDNA clone MGC:89716 IMAGE:7026875), complete cds

The four most abundant gene families of globins, mitochondrial encoded proteins, proteins associated to the actin-myosin cytoskeleton, and ribosomal proteins comprised 33.5% of all ESTs represented by 23 contigs.

In order to subtract our dataset from contig sequences that are already available for *N. viridescens *in NCBI databases, we performed BLAST searches applying a cut-off value of ≤ e-25. We identified a significant homology to 30 NCBI nr entries, which represented 19 different proteins. 12 out of these 19 proteins were encoded on the mitochondrial DNA (Table 3). Most of the known *N. viridescens *NCBI entries were not covered by our EST data set, which might be due to a low expression level and/or the different tissues from which the ESTs were derived. In summary, we were able to expand the existing set of unique NCBI sequence entries by more than 1600 high quality annotated contigs, of which 174 sequences represented putative proteins that are potentially unique to urodele amphibians.

**Table 3:** Matches of newt heart ESTs to existing NCBInr entries for *N. viridescens*.

Description	Accession No	**Match to con**.	E-value
NADH dehydrogenase subunit 1 [Notophthalmus viridescens]	gi|213136180; gb|ACJ43727.1	883	< 1e-150

NADH dehydrogenase subunit 1 [Notophthalmus viridescens]	gi|13561422; gb|AAK30304.1	883	< 1e-150

NADH dehydrogenase subunit 2 [Notophthalmus viridescens]	gi|213136181; gb|ACJ43727.1	119	< 1e-150

NADH dehydrogenase subunit 2 [Notophthalmus viridescens]	gi|13561421; gb|AAK30303.1	119	< 1e-150

cytochrome c oxidase subunit 1 [Notophthalmus viridescens]	gi|213136182; gb|ACJ43728.1	47 2245	< 1e-1508e-096

cytochrome c oxidase subunit 1 [Notophthalmus viridescens]	gi|13561423; gb|AAK30305.1	47	2e-009

cytochrome oxidase subunit I [Notophthalmus viridescens]	gi|156788427;gb|ABU95787.1	47 2245	< 1e-1501e-108

cytochrome c oxidase subunit 2 [Notophthalmus viridescens]	gi|213136183; gb|ACJ43729.1	47 2096 2909 2230	< 1e-150< 1e-1501e-104 5e-049

ATP synthase F0 subunit 8 [Notophthalmus viridescens]	gi|213136184; gb|ACJ43730.1	104	3e-059

ATP synthase F0 subunit 6 [Notophthalmus viridescens]	gi|213136185; gb|ACJ43731.1	104 2301	< 1e-1506e-027

cytochrome c oxidase subunit 3 [Notophthalmus viridescens]	gi|213136186; gb|ACJ43732.1	55 2933	< 1e-150< 1e-150

NADH dehydrogenase subunit 3 [Notophthalmus viridescens]	gi|213136187; gb|ACJ43733.1	483	< 1e-150

NADH dehydrogenase subunit 4 [Notophthalmus viridescens]	gi|213136189; gb|ACJ43735.1	498	< 1e-150

NADH dehydrogenase subunit 4 [Notophthalmus viridescens]	gi|58202527; gb|AAW67315.1	498	< 1e-150

NADH dehydrogenase subunit 5 [Notophthalmus viridescens]	gi|213136190; gb|ACJ43736.1	428	< 1e-150

NADH dehydrogenase subunit 6 [Notophthalmus viridescens]	gi|213136191; gb|ACJ43737.1	428	< 1e-150

cytochrome b [Notophthalmus viridescens]	gi|213136192; gb|ACJ43738.1	2181	< 1e-150

cytochrome b [Notophthalmus viridescens]	gi|58202439; gb|AAW67271.1	2181	< 1e-150

cytochrome b [Notophthalmus viridescens]	gi|75858084; gb|ABA28616.1	2181	< 1e-150

cytochrome b [Notophthalmus viridescens]	gi|75858082; gb|ABA28615.1	2181	1e-114

cytochrome b	gi|529472; gb|AAA31972.1	2181	1e-105

cytochrome b	gi|529470; gb|AAA31971.1	2181	4e-054

histone H3a [Notophthalmus viridescens]	gi|90185040; gb|ABD91461.1	925	6e-025

Newt histone H4 gene, partial	J00954.1; GI:213407	2887	4e-046

Tubulin alpha chain	Q91060; GI:3024695	1759 1820 1653	< 1e-150< 1e-1502e-029

alpha-tubulin [Notophthalmus viridescens]	gi|468544; emb|CAA83457.1	1759 1820 1653	< 1e-150< 1e-1502e-029

cytokeratin type II [Notophthalmus viridescens]	gi|2370339; emb|CAA04655.1	145	5e-026

cytokeratin 8 [Notophthalmus viridescens]	gi|2370339; emb|CAA04655.1	145	< 1e-150

complement component C3 [Notophthalmus viridescens]	gi|28372361; gb|AAO38043.1	2286	1e-103

tissue inhibitor of metalloproteinase 1 [N. viridescens]	gi|82659076; gb|ABB88702.1	952	< 1e-150

Significant similarities to the newt EST database were obtained for 30 entries from the NCBInr database. Most contigs matched to mitochondrial-encoded proteins

### Functional annotation of assembled newt contigs to Gene Ontology terms

In order to achieve a functional annotation of assembled newt contigs we took advantage of protein identifiers deposited in the GOA database. Protein identifiers were retrieved by searching the TrEMBL and Swiss-Prot databases using e-value ≤ e-20 and an Abagyan score of >15 [22]. Using this approach we assigned 1116 contigs to existing GO terms. 1043 GO terms were assigned to the domain "biological process", 1063 GO terms to "molecular function" and 1045 GO terms to "cellular component". 71.11% of all functionally annotated proteins were most similar to the mammalian species human, mouse, rat and bovine, 11.02% to fungal proteins, 10.85% to insects, whilst 6.35% displayed the highest similarity to miscellaneous eukaryotic organisms. Only 0.67% of all GO annotated proteins were assigned to Archaebacteria and bacteria (Table 4).

**Table 4:** Distribution of GO annotated protein identifiers to different animal classes.

Protein Identifier	Total	Ratio [%]
All Organisms	1116	100

Mammalian	554	49,64

Amphibian	356	31,90

Avian	86	7,70

Fish	80	7,17

Insects	13	1,16

Prokaryotes, virus	9	0,80

Unicellular Eukaryotes	7	0,63

Reptiles	4	0,36

Other invertebrates	4	0,36

Plants	3	0,28

50% of best hits matched to mammalian organisms. Only 3.59% of all best hits matched to insects, lower vertebrates, lower eukaryotic organisms, and bacteria.

### Comparison of transcriptional changes during newt and zebrafish heart regeneration

Cardiac regeneration in vertebrates does not only occur in the newt *N. viridescens *but also in zebrafish [23]. The availability of Affymetrix DNA microarrays for zebrafish, which covers around 15000 transcripts, allowed an analysis of the gene expression profile during zebrafish heart regeneration [24]. In this analysis 662 ESTs were found to change their expression level during heart regeneration. We wondered whether our newt cDNA library, which was constructed from regenerating hearts, would cover corresponding sequences from zebrafish. To answer this question we integrated available zebrafish cDNA sequences into a database. Analysis of the database using the published Affymetrix data allowed us to extract deregulated zebrafish transcripts, which we assembled into 632 different contigs. We next searched our newt EST library with deregulated zebrafish sequences yielding 70 BLAST hits with an e-value < e-05. Of these hits, 46 individual newt contigs were identified, which showed a similarity to 50 zebrafish Affy-IDs (Table S2 in Additional file 3). Zebrafish and newts are evolutionary distant organisms. This compromises comparisons on the nucleotide level by BLAST and restricts identification of potential homologous protein sequences. Furthermore, the transcriptomes of zebrafish and from newts are far from being complete. It is likely that EST clones from newts and zebrafish, which encode for a specific homologous protein, will not match since the clones encode different non-overlapping parts of the cDNA. To account for these difficulties we assigned protein identifiers to newt and zebrafish EST clones after BLASTx searches on TrEMBL and Swiss-Prot databases. In total, 284 zebrafish sequences were linked with a protein identifier. 31 proteins shared identical identifiers with proteins from our newt dataset, which were not detected previously by direct BLAST searches (Table 5). Using this approach we detected additional EST clones coding for raldh2 in both newt and zebrafish data sets. Raldh2 is a marker of activated epicardial cells that cover damaged myocardium after amputation and migrate actively into the regenerating myocardium [25]. Applying both direct BLAST searches and comparison of protein identifiers we were able to identify similarities to 77 of the 284 deregulated zebrafish proteins. This indicates that a substantial number of the 1116 annotated newt proteins may change expression levels during heart regeneration.

**Table 5:** List of identical protein identifiers from deregulated zebrafish proteins and newt proteins annotated to TrEMBL and SwissProt databases.

Entry name	Organism	UniProtKB/SwissProt entry
ACTN4	MOUSE	P57780

ADSV	MOUSE	Q60604

ADT3	HUMAN	P12236

AL1A2	HUMAN	O94788

Q804G7	DANRE	Q804G7

APOEB	DANRE	O42364

BIRC5	HUMAN	O15392

CHERP	HUMAN	Q8IWX8

CHK1	XENLA	Q6DE87

CLUS	MOUSE	Q06890

CO5A3	HUMAN	P25940

CP18C	ARATH	P34790

CTGF	MOUSE	P29268

FINC	HUMAN	P02751

HSP72	RAT	P14659

HSP7C	MOUSE	P63017

HSP7D	DROME	P11147

HSP83	DROME	P02828

K2C1	HUMAN	P04264

K2C5	BOVIN	P13647

K2C8	MOUSE	P11679

KCRS	HUMAN	P17540

MYBPH	MOUSE	P70402

PLK1	MOUSE	Q07832

PPIA	DROME	P25007

Q1L9G7	DANRE	Q1L9G7

TBB3	DROME	P08841

TBB5	HUMAN	P07437

TENA	MOUSE	Q80YX1

TENR	MOUSE	Q8BYI9

TYB	DANRE	Q9W7M8

Identifiers with best hits are listed with protein name, corresponding organism, and UniprotKB/swissprot entry. Additional 31 proteins were identified using this approach.

**Figure 3:**
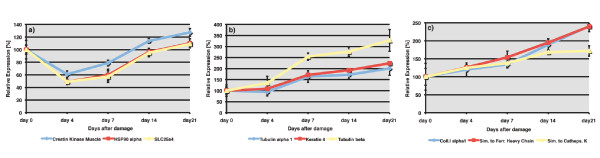
**Gene expression profile of selected newt-zebrafish homologues**. Quantification of gene expression patterns by RT-PCR of potential newt-zebrafish homologues during newt heart regeneration in undamaged (day 0) and damaged newt hearts 4, 7, 14, and 21 days (day 4 to day 21) after injury (n = 3 for each time point). a) Expression of muscle creatin kinase, hsp90 alpha, and slc25a4. Potential zebrafish homologues showed a similar expression pattern although reduced mRNA levels were observed at 4 days to 14 days after injury. b) Expression of tubulin alpha 1, keratin 4 and tubulin beta. A delayed time course was apparent in regenerating newt hearts with increased levels only at 21 days after injury. c) Expression of collagen typeI alpha 1, similar to ferritin heavy chain, and similar to cathepsin K. Similar changes in expression levels were found both in newts and zebrafish at all time points after injury. Expression changes of more than two fold were statistically significant with the exception of tubulin 1 alpha at 21 days after injury (p < 0.05 by paired students t-test). Error bars are shown as ± STDEV.

To further investigate whether both organisms show similar changes in the gene expression program during heart regeneration we performed RT-PCR analyses of selected candidate genes categorized into 3 different gene expression clusters (cluster I, III and V) according to Lien et al 2006 [25]. 3 genes of each selected cluster were chosen. Expression levels were determined in newt hearts without injury and at 4, 7, 14 and 21 days after mechanical damage. For each time point analyzed, 3 biological sample pools, consisting of 4 newt hearts each were used. We found that Muscle Creatin Kinase, HSP90 alpha and SLC25a4, which belong to cluster I, were down regulated by about 50% in newt hearts 4 days after damage. The expression was similar to control levels at day 14 and 21 after damage. In zebrafish these genes showed an extended down regulation 3, 7 and 14 days after amputation, which might be due to the more severe type of injury (Figure 3a). Genes of cluster III (Tubulin alpha1, Keratin 4 and Tubulin beta2) were up regulated 3 and 7 days after zebrafish ventricular amputation while the expression levels of corresponding newt sequences were not elevated at day 4 but increased from day 7 onwards until 21 days after damage. The delayed up-regulation of cluster III genes in newts together with the extended increase of expression levels was clearly different from the situation in zebrafish (Figure 3b). The genes for Collagen typeI alpha1, similar to Ferritin Heavy chain and similar to Cathepsin K (cluster V) were up regulated at all stages of newt and zebrafish heart regeneration, albeit with different kinetics. In zebrafish, the highest levels occurred at earlier stages during development while this pattern was inversed in regenerating newt hearts (Figure 3c). All expression changes of more than two fold were analyzed for statistical significance by paired students t-test and were found to be significant (p < 0.05). The only exception was the expression of tubulin 1 alpha at 21 days after injury. Taken together our analysis revealed a significant change in expression level of members of corresponding gene families in regenerating hearts of zebrafish and newt. The time course of expression, however, differed between both species, which might reflect species-specific differences or the different types of injuries, which were applied.

### Identification of biological pathways activated during zebrafish and newt heart regeneration by GO annotation

So far the analysis indicated that our EST dataset is enriched in sequences, which were modulated during newt heart regeneration, and that such sequences were also often expressed at different levels during regeneration of zebrafish hearts. We next asked whether we could identify common transcriptional pathways or signaling cascades, in which annotated newt proteins accumulate. We assumed that deregulated zebrafish ESTs represent a subset of genes, which mark regenerative processes. All newt and zebrafish proteins that were previously assigned to a GO term within the root node "biological process" (1043 and 244 protein sequences, respectively) were included in our search for genes involved in regeneration. We then selected a number of daughter nodes that cover processes, which are most likely involved in tissue regeneration. The following nodes were selected: 1.) "Embryonic or larval gene expression programs" since regeneration might require the re-initiation of early embryological processes which are needed to build tissues or organs. 2.) " Wound healing and cell migration", since the early phase of heart regeneration in zebrafish and newt involves recruitment and migration of inflammatory cells into the damaged tissue and other typical wound healing processes [4,23]. 3.) "Cell proliferation and cell death", since remodeling of damaged tissue requires proliferation of cells and removal of non-functional cell types within the damaged heart. 4.) "Cell differentiation, cell division and cell cycle", since new functional cells have to be generated by cellular differentiation of precursor cells that might originate from de-differentiated, reprogrammed cardiomyocytes [2,13,26] or from stem cells residing within the heart [25]. 5.) "Circulation and growth of tissue", since epicardial cells migrate into the wound area of zebrafish hearts and contribute to the establishment of functional cardiac tissue by formation of microcapillaries [25]. 6.) "Muscle contraction", since reprogramming of existing newt cardiomyocytes results in down regulation of mature cardiomyocyte markers and expression of smooth muscle proteins [13]. 7.) "Cell surface receptor linked signal transduction and intracellular receptor mediated signaling pathways", since signaling events, which originate at the cell membrane, will contribute to the initiation and regulation of cardiac regeneration.

We first generated a tree diagram displaying all possible GO term daughter nodes derived from the above selected ancestor terms using the freeware tool BLAST2GO [27] (Figure S3 in Additional file 4). Next, proteins were directly annotated to their respective GO nodes and then annotated to their connected parental nodes until the root biological process was reached. This allowed us to functionally connect proteins that are otherwise not assigned to the same specific GO term. 599 protein identifiers were directly associated to 331 different GO terms in newts (Table 3 in additional file 5). For the zebrafish dataset we associated 192 protein identifiers to 197 GO terms (Table S3 in additional file 5). The largest number of protein identifiers (almost one third of all annotated newt proteins and 30% of all zebrafish proteins) was related to a GO term connected to development. A clear difference in protein numbers between newt and zebrafish was detected for the GO ancestor term "intracellular receptor mediated signal", which is most probably due to relative small numbers of proteins associated with this term in the zebrafish dataset. Interestingly, we did not detect any protein identifier for the ancestor term "antigen receptor-mediated signaling pathway" in zebrafish and only 6 proteins within the newt database suggesting that cellular immune response may be of minor relevance at these stages of heart regeneration.

The total number of proteins within a dataset that has been assigned to a given GO term does not necessarily reflect the relative distribution of proteins between different terms, since the number of proteins that are covered by a given term might differ. Therefore, we compared the total number of proteins annotated to the GO term biological process to the total number of proteins in selected GO term nodes. This procedure allowed us to determine the ratio of proteins annotated to the GO term nodes GO:0007275: development, GO:0042060: wound healing, GO:0016477:cell migration, GO:0008283: cell proliferation, GO:0008219: cell death, GO:0030154: cell differentiation, GO:0051301: cell division, GO:0007049: cell cycle, GO:0000278: mitotic cell cycle, GO:0008015: circulation, GO:0040007: growth, GO:0006936: muscle contraction and GO:0007166: cell surface receptor linked signal transduction. To verify an accumulation of proteins in a given ancestor term, we also compared the ratio of proteins annotated to a given GO term to the total number of proteins available from the GOA database, which was used as an unbiased standard dataset (Table S3 in additional file 5).

**Figure 4:**
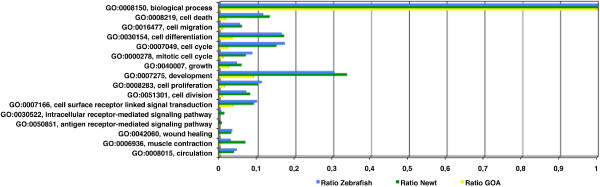
**Ratio of proteins annotated to selected ancestor GO terms relative to all proteins annotated to the GO term biological process for newt, deregulated zebrafish proteins, and protein entries from the complete GOA database**. A more than 10 fold accumulation in newt and zebrafish EST datasets was detected within the GO term nodes wound healing, muscle contraction and circulation.

Interestingly, we found a strong accumulation of proteins in some of the selected ancestor terms. A more than 10 fold accumulation was detected for "wound healing", "muscle contraction", and "circulation". The GO terms "cell division", "cell death", "cell migration", "cell cycle", "mitotic cell cycle" and "cell proliferation" displayed a fold enrichment of at least 6.27 for zebrafish and 6.5 for newt with a minimum of 13 proteins annotated for zebrafish and 61 proteins annotated for newt. The terms "cell differentiation", "growth" "development", and "cell surface receptor linked signal transduction" showed an enrichment of less than 5 fold, which confirms our starting hypothesis that the total number of proteins, which can be linked to a GO term, does not necessarily reflect the relative activity of genes within a term (Figure 4).

### Identification and analysis of genes associated with highly enriched GO term sub nodes

**Figure 5:**
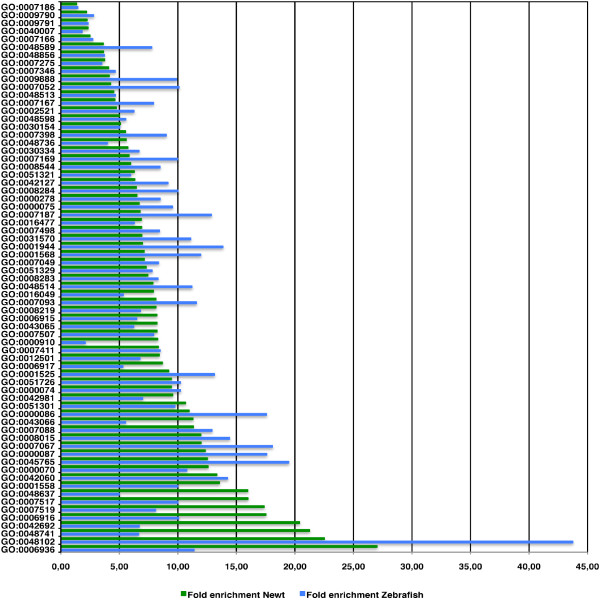
**Accumulation of proteins annotated to GO terms containing more than 2 zebrafish proteins and more than 7 newt proteins**. Fold enrichment was determined by comparing the ratio of proteins annotated to the complete GOA dataset relative to the ratio of annotated newt and zebrafish proteins. 12 GO term nodes display more than 10 fold enrichment in proteins for newt and zebrafish datasets.

Since the determination of protein numbers accumulating within one of the above described ancestor terms only gives an estimate which general categories of a biological process were activated, we next determined the fold enrichment of proteins in all 331 GO sub nodes for newt and all 192 GO sub nodes for zebrafish. To demonstrate that this procedure allows identification of genes, which are not necessary homologous but linked to similar biological processes, we selected daughter nodes of GO terms that contained at least 3 protein identifiers from zebrafish and 8 protein identifiers for newt (Figure 5 and table S3 in additional file 5). We detected an at least 10-fold enrichment for the following 12 GO term nodes (the numbers in brackets indicate the total number of proteins in the newt and zebrafish datasets): M phase of mitotic cell cycle (54, 18), mitosis (51, 18), regulation of mitosis (15, 4), mitotic sister chromatid segregation (20, 4), and G2/M transition of mitotic cell cycle (8, 3). The enrichment within these sub nodes indicated a dynamic regulation of the cell cycle during cardiac regeneration; anti-apoptosis (51, 7), and autophagic cell death (11, 5) suggesting a major role of the inhibition of programmed cell death and removal of dysfunctional cells during regeneration; muscle development (82, 12), and muscle contraction (72,7) as expected for a dataset derived from contractile tissue; circulation (39, 11), regulation of angiogenesis (11, 4), and wound healing (32, 8), which demonstrated the critical role of neoangiogenesis and wound repair during early heart regeneration.

To prove that enrichment analysis of GO terms allows a more efficient identification of deregulated candidate genes that might be involved in the regeneration process rather than random selection, we performed statistical analysis for all 331 GO terms from the newt dataset based on chi square test. In the course of the analysis we made a one-to-one comparison for the GOA and newt datasets. The p-values represent the level of significance by which our observations are independent from each other. For the above-mentioned GO terms, the detected enrichment was statistically significant (Table S4 in additional file 6).

To learn more about the expression kinetics of individual genes which were present in highly enriched GO terms (>10-fold), we performed a RT-PCR analysis using tissues from hearts isolated at four different time points during heart regeneration. Four genes were selected that were both identified in newt and zebrafish hearts: 1.) Karyopherin or importin alpha2 (kpna2, importin alpha-2), which is involved in the first step of nuclear protein import and is believed to be regulated in a cell cycle dependent manner [28]. Karyopherin is regulated by TGF beta1 and IFN-gamma mediated signaling [29] amongst many other pathways. 2.) Survivin (Baculoviral IAP repeat-containing protein, BIRC5), which is member of a family of inhibitors of apoptosis and functions as a key regulator of mitotic cell cycle and programmed cell death [30]. Survivin is a target of the PI3K/Akt Pathway [31] and interacts in a cell cycle dependent manner with the small GTPase Ran [32]. 3.) Clusterin (Apolipoprotein J, Apo-J), which is a glycoprotein expressed as an intracellular isoform, and as a secreted protein. The nuclear form of clusterin (nCLU) is pro-apoptotic while the secretory form (sCLU) has a pro-survival role [33,34]. Clusterin has been implicated in various cell functions involved in carcinogenesis and tumor progression [35]. 4.) Cyclophilin A (Peptidyl prolyl isomerase A, PPIA), which is a member of the peptidyl-prolyl cis-trans isomerase (PPIase) family involved in cyclosporin A mediated immunosuppression [36,37]. In H1299 lung cancer cells, down regulation of Cyclophilin A prevents up regulation of Cyclin D1 and cdk4 expression for subsequent cell cycle progression [38]. The expression level of all 4 selected genes differed from normal hearts at all 4 time points analyzed. The highest expression was detected at 21 days after injury. An almost two fold change was detected for survivin, which was down regulated to less than 60% of normal levels 4 days after newt heart damage, although changes in expression levels were statistically not significant (data not shown). The expression of survivin in zebrafish was up regulated with expression levels that were highest at 3 and 7 days after amputation. This illustrates differences during newt and zebrafish heart regeneration despite multiple similarities.

To ask whether our GO term based functional screen is able to identify additional transcripts that may change their expression level during newt heart regeneration, we selected candidate proteins from GO terms, which showed a more than 10 fold accumulation ratio. Candidates were chosen which either did not undergo a change in expression levels during zebrafish heart regeneration or which were not present on zebrafish Affymetrix arrays. For transcripts associated to "cell cycle regulation" we included the GTP-binding nuclear protein Ran, which is required for nucleo-cytoplasmic shuttling of proteins and control of DNA synthesis and cell cycle progression [39,40]. Ran directly interacts with survivin in a cell cycle dependent manner [32]. RING-box protein 1, a component of the SCF (SKP1-CUL1-F-box protein) and the CBC (VHL) (CUL2-elonging BC-VHL) E3 ubiquitin ligase complexes mediates ubiquitination and subsequent proteasomal degradation of target proteins involved in cell cycle progression [41,42]. Thioredoxin-like protein 4B (Dim1), which is required for cell cycle progression at the S/G(2) transition and is associated to proteins involved in pre-mRNA splicing [43,44]. DEAD/H box protein 11 (Chlr1), a DNA helicase required for sister chromatid cohesion [45]. Chlr1 is essential for embryonic development and the prevention of aneuploidy [46]. S-phase kinase-associated protein 1, an essential component of the SCF (SKP1-CUL1-F-box protein) ubiquitin ligase complex [47], which mediates the ubiquitination of proteins involved in cell cycle progression. Fission yeast skp1 is required for spindle morphology and nuclear membrane segregation at anaphase [48]. Aurora kinase A-interacting protein (AKIP), a negative regulator of Aurora-A kinase [49], that modulates the stability of its interaction partner Aurora-A kinase in conjunction with GSK-3beta [50], as well as Centrin 2 and 3, involved in centrosome reproduction [51,52]. Additionally, we selected transcripts assigned to negative regulation of apoptosis. Translational-controlled tumor protein (TCTP1, Fortilin), a multifunctional protein with potent antiapoptotic capabilities [53] that is essential for mouse embryonic development [54]. Secreted frizzled-related protein 1 (SFRP1), which functions as a modulator of Wnt signaling through direct interaction with Wnts [55]. Ring finger protein 7 (RNF7, RBX2, SAG) a component of the SCF (SKP1-CUL1-F-box protein) E3 ubiquitin ligase complex, which mediates ubiquitination and subsequent proteasomal degradation of target proteins involved in cell cycle progression, signal transduction and transcription [56]. High mobility group box protein B1, which functions as an intracellular regulator of transcription and is also released as a cytokine by necrotic and inflammatory cells [57]. HMGB-1 is implicated to be critically involved in skeletal muscle [58], as well as cardiac regeneration [59]. Cofilin 2, an actin depolymerizing protein, implicated in congenital Nemaline myopathy (NM) [60]. PP1beta, potentially involved in cell cycle control and apoptosis [61] and YWHAZ (14-3-3- zeta), which possibly modulates reorganization of the actin cytoskeleton via binding to cofilin and LIMK1 [62]. From a third group of transcripts functionally annotated to muscle development, we chose Muscle LIM protein (CSRP3), which is critically involved in cardiomyocyte architecture, as revealed by targeted knockout [63]. Muscle LIM protein acts as a stress sensor, linked to calcineurin-NFAT signaling at the sarcomeric Z-disk [64] and Interferon-related developmental regulator 1 (IFRD1), a multifunctional transcriptional regulator, that is directly interacting with p53, regulating its function [65] and is also required for myoblast differentiation [66].

**Figure 6:**
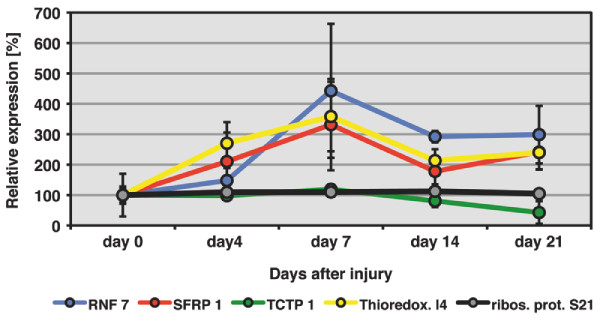
**Expression profiles of selected mRNAs during newt heart regeneration**. Expression of RNF 7, SFRP 1, Thioredoxin-like protein 4B and TCTP 1 was analysed by RT-PCR at 4, 7, 14 and 21 days after mechanical injury of newt ventricles (n = 3 for each time point). Expression of ribosomal protein S21 was analyzed as a non-modulated control. A more than 2 fold change in expression level was detected for all 4 selected genes during the newt heart regeneration. Statistically significant changes in expression (p < 0.05 by paired students t-test) were detected for RNF 7 and SFRP 1 at 14 and 21 days after injury and 7 and 14 days after injury for Thioredoxin-like protein 4B. Error bars are shown as ± STDEV. Please note that selected newt genes were so far not identified in regenerating zebrafish hearts.

To detect transcriptional changes during newt heart regeneration, we performed RT-PCR analysis for the selected candidates as described above. Although most candidate genes did not show major changes in expression, we monitored a more than 2 fold change in the levels of RNF 7, SFRP 1, Thioredoxin-like protein 4B and TCTP 1 during newt heart regeneration (Figure 6). These changes were statistically significant for RNF 7 and SFRP 1 at 14 and 21 days after injury and 7 and 14 days after injury for Thioredoxin-like protein 4B (p < 0.05 by paired students t-test). We concluded that our functional annotation based screen identified dynamically regulated candidate genes that have not been implicated in cardiac regeneration before. Although a functional analysis is pending, the changes in the expression of cell cycle control genes and of regulators of programmed cell death suggest an important role of these processes for newt heart regeneration.

## Conclusion

We have described the generation of a large EST dataset from regenerating newt hearts. This will facilitate the molecular analysis of heart regeneration. Together with existing EST data from other urodeles such as *Amybystoma mexicanum*, our EST dataset might also help to uncover general principles of tissue regeneration in urodele amphibians. Careful analysis of our data allowed insights into the transcriptional program that governs heart regeneration in newts and revealed multiple changes in different biological processes ranging from the regulation of programmed cell death to cell cycle regulation and wound repair. Comparison to sequences, which are deregulated during zebrafish heart regeneration, revealed similarities but also differences during cardiac regeneration in both species.

The fundamental differences between mammals and newts in tissue regeneration argue for involvement of multiple mechanisms directing complex processes ranging from wound repair, regulation of the extracellular matrix, and cell cycle control, to proliferation and differentiation, which need to be precisely controlled to allow successful organ regeneration. Only integrated approaches that take advantage of the analysis of large data sets will eventually lead to a thorough understanding of tissue regeneration. An important part of such analysis is the comparison between organisms capable of comprehensive organ regeneration and those, which have lost (or suppress) this ability. So far the mammalian heart has proven to be resistant to tissue regeneration despite numerous attempts to rebuild lost tissue including cell transplantation and activation of potential regenerative pathways. It seems reasonable to assume that analysis of transcriptional changes, which occur in organisms capable of regeneration, might disclose the critical processes that are indispensable for the replacement of functional heart tissue. We reason that in-depth knowledge of differences that occur in response to tissue damage between regenerating and non-regenerating organisms might pave the way to manipulate and improve regenerative responses. Eventually, evolutionarily imposed restrictions to cardiac regeneration might be lifted, in particular if such restrictions are species-specific. The acquisition of sequence data, which reflect transcriptional changes during newt heart regeneration, is a first step in this direction.

## Methods

### Animal housing and surgery

All newts were obtained from Charles D. Sullivan newt farm, Nashville Tennessee. Adult animals were kept in aerated fresh water aquaria at constant temperature between 18-20°C and were fed twice a week with artemia larvae. For surgery, newts were deeply anaesthetized with 0.1% by Ethyl-3-aminobenzoate-methanesulfonic acid salt solved in water. Skin and body wall were opened by an incision; hearts were gently pulled out, and fixed with fine forceps at the ventricle. Injury was done be squeezing the ventricle for 15 times each in two directions, avoiding extensive bleeding. After repositioning of hearts, incisions were closed with Histoacryl (B. Braun Aesculap AG, Germany). Newts were kept for several hours in 0.5% sulfamerazine solution to avoid infections. Operated newts were kept in separate aquaria under constant observation. To collect heart samples, animals were deeply anesthetized decapitated and hearts were immediately removed and flash frozen in liquid nitrogen.

### cDNA library construction, sequencing and EST contig assembly

Total RNA was isolated using Trizol according to the manufacturers instructions (Invitrogen). To construct the cDNA library 1 μg of polyA^+ ^was purified from 30 newt hearts and subjected to the Creator SMART kit protocol as described by the manufacturer (Clontech). Briefly, 1 μg of polyA^+ ^RNA was used to synthesize first strand cDNA. Double stranded cDNA was synthesized by primer extension, digested with SfiI, size fractionated and ligated into pDNRLib vector (Clontech). Clones were transformed into E. coli, plated onto LB/Agar plates, containing 25 μg/ml Chloramphenicol and arrayed into 384 well plates (RZPD, Berlin, Germany). Sequencing of 11520 EST clones was performed at the Max-Planck-Institute of Molecular Cell Biology, Dresden. Trace data of minor quality were removed and sequences were clipped from contaminating vector information and subsequently from 3' polyA tails. Inserts below 100 bp in length, and clones, not containing cDNA inserts were subtracted and discarded. The resulting set of 9698 EST sequences served as an input for all further analysis. Assembly was performed with the commercial software SeqMan™ Pro (DNASTAR, Lasergene) using default parameters.

### BLAST search, functional annotation, EST submission and identification of enriched GO terms

BLAST searches for 2894 contigs were performed on NCBI nr and EST databases with blastn, blastx and tblastx algorithms. BLAST output files were parsed with a cut-off E-value = e-05. For each contig, 50 top scoring BLAST hits were stored in the database. Functional annotation to Gene Ontology terms was performed via BLASTx searches on TrEMBL and Swiss-Prot databases with an e-value cut-off < e-20 or Abagyan score higher than sigma = 8 [22]. Protein identifiers were derived for 50 top scoring hits, independent of organism according to the order of BLAST hit ranking. Identifiers were used to attach GO terms from the GOA database. For contigs with no assignable similarity (e-value > 1-05), potential open reading frames and 3'untranslated regions were searched with public available software ESTscan [21]. Annotation to GenBank was performed by splitting 2894 contigs into their respective ESTs, that were attached with the information of top ranking hits (score ≤ e-05) from all blast algorithms performed. We defined an e-value smaller than 1e-20 as "significant similarity" and an e-value between 1e-20 and e-05 as "weak similarity.

The total number of annotated proteins for newt and zebrafish datasets and all proteins within the GOA database (as of October 2008) were calculated for selected GO terms and used to determine the ratio of the total number of annotated proteins within the GO term "biological process" for all three databases used. The degree of enrichment against the GOA dataset was expressed as fold accumulation.

### Annotation of deregulated Zebrafish Affy IDs

Deregulated zebrafish EST clones were selected from the Affymetrix™ zebrafish microarray sequence data file [24]. Corresponding sequences were assembled using the commercial SeqMan™ Pro software to yield 632 contigs. Contig annotation was done using the blastx, blastn and tblastx algorithms against NCBI nr protein, nr nucleotide and EST databases using a similarity cut-off < 1e-20 (= "significant similarity") and a cut-off ≤ 1e-05 (= "weak similarity"). Assignment of GO terms was performed in the same manner as with newt contigs using blastx searches against Swiss-Prot and TrEMBL protein databases. BLAST searches against the newt EST database were performed using BLASTn with all 662 deregulated zebrafish ESTs with an e-value cut-off smaller than 1e-05.

### RT-PCR Analysis

For each time point analyzed (uninjured heart, 4, 7, 14, and 21 days after injury), 3 biological samples, pooled from 4 individual newt hearts each, were used for RNA isolation with Trizol reagent (Invitrogen). 1 μg of total RNA was reverse transcribed with Superscript II™ (Invitrogen) in a total volume of 50 μl. Oligonucleotides for all 30 candidate genes are listed in additional file 7. PCR was performed with Taq-Polymerase in 25 μl reactions at 55°C for 25-35 cycles. PCR products were visualized by agarose gel electrophoresis. Band intensities were analyzed densitometrically with Image J Version 1.40 g and used to calculate mRNA expression levels.

### Data storage in a web based relational database

All information was stored in a relational database (MySQL) to facilitate easy access to the data via a web browser independent PhP frontend. The EST database was constructed as an organism unspecific relational database using the open source software MySQL. The web-based frontend was realized in PHP, database update pipelines were programmed in Perl. The open source software "apache" was used for web server services. The design of the database allows storing of newt sequence data, such as EST sequences, their respective plate coordinates, assembly information, and all annotation data derived from NCBI BLAST searches in nr and EST databases with its associated alignment and e-value score (Figure S4a in Additional file 8). It is possible to search for cDNA library plate coordinates via the frontend as well as for assembled contigs by contig number, assigned accession, or description (data not shown). Moreover, similar proteins derived from TrEMBL and Swiss-Prot databases as well as resulting functional annotation of these proteins from the GOA database were stored together with the e-value and abagyan score and can be searched via the frontend (Figure S4b in Additional file 8). All stored identifiers are directly linked to external public access databases for advanced analysis. To allow coherent GO term analysis the entire GO term tree was deposited and is browseable via the frontend, which displays the degree of direct and indirect enrichment in single GO nodes (figure S4c in Additional file 8). Further GO term visualization was implemented by links to external GO term viewers (EMBL-EBI QuickGO and SRS). The GO terms are accessible with search forms for GO term identifiers, GO term descriptions and protein descriptions. The database provides changeover between GO term-, assigned proteins- and assembled contigs-view (data not shown). The frontend does also house a BLAST search form, which allows BLASTN and BLASTX searches within the EST database for analysis of additional sequences (Supplementary figure S4d in Additional file 8). We also included an update form to facilitate addition of new sequences with corresponding contigs and annotation data. The database can be used as an annotation viewer for external software like Acuity (Axon Instruments) by a PhP based interface. The database scheme is independent of organisms. Based on a set of sequences, a pipeline of scripts generates all required data and formats relational tables. For the comparison with deregulated zebrafish genes, we also generated a zebrafish database. An updated version of the web-based frontend of the newt EST database will be publically available as of December 2009.

## Authors' contributions

TBo designed the study, TBo and TBr wrote the manuscript, TBo performed the expression analysis and GO enrichment analysis. ML performed in silico EST analysis, contig assembly, BLAST searches, homology assignments and EST annotations. ML and PW programmed the database interface and web-based frontend. PW programmed the functional annotation to GO terms tool. MB performed the statistical significance analysis for GO term enrichment. JK cloned the cDNA library. All authors read and approved the final manuscript.

## Supplementary Material

Additional file 1 **2 supplementary figures**. Figure S1 shows the read length distribution of 9696 high quality ESTs. Figure S2 shows the length of 2894 contigs and the statistics of ESTs per contig. Click here for file

Additional file 2 **Best Blast hits to 1695 contigs with score ≤ 1e-05**. Best BLAST hits for each contig were extracted from 5 different BLAST algorithms, sorted by contig ID, BLAST algorithm, and database hit, description and corresponding e-value. Click here for file

Additional file 3 **BLAST results of 662 deregulated zebrafish ESTs to the newt heart EST database**. 50 zebrafish affymetrix IDs were matched to 46 individual newt contigs with an E-value < 1-05. Click here for file

Additional file 4 Figure S3 shows a graph of GO term nodes used to functionally annotate newt and zebrafish proteins. Click here for file

Additional file 5 **Numbers of newt and zebrafish proteins annotated to preselected sub nodes of GO:0008150: biological process**. To the GO term sub nodes GO:0008219: cell death, GO:0016477: cell migration, GO:0030154: cell differentiation, GO:0007049: cell cycle, GO:0000278: mitotic cell cycle, GO:0040007: growth, GO:0007275: development, GO:0008283: cell proliferation, GO:0051301: cell division, GO:0007166: cell surface receptor linked signal transduction, GO:0030522: intracellular receptor-mediated signaling pathway, GO:0050851: antigen receptor-mediated signaling pathway, GO:0042060: wound healing, GO:0006936: muscle contraction and GO:0008015: circulation, the number of proteins within daughter nodes was determined for newt and zebrafish and compared to the number of proteins deposited in the GOA database. The ratio of abundance was determined by dividing the number of annotated proteins within a daughter node to the total number of proteins annotated to GO: 0008150: biological process. Click here for file

Additional file 6 **Statistical analysis of GO term enrichment**. Chi-square test for one to one observations from the newt dataset against the GOA dataset. Level of significance is given as p-value. Click here for file

Additional file 7 List of oligonucleotides used for RT-PCR analysis from regenerating newt hearts. Click here for file

Additional file 8 **Figure**. Figure S4 demonstrates the features of the newt database. Click here for file
