# Supplementary material for: Analysis of newly established EST databases reveals similarities between heart regeneration in newt and fish
Source: BMC Genomics. 2010 Jan 4;11:4. doi: 10.1186/1471-2164-11-4 (PMC2823690; doi:10.1186/1471-2164-11-4)
Supplement: Additional file 1 — 2 supplementary figures. Figure S1 shows the read length distribution of 9696 high quality ESTs. Figure S2 shows the length of 2894 contigs and the statistics of ESTs per contig. [file 1471-2164-11-4-S1.PDF]

Borchardt et al., Supplementary Figure 1

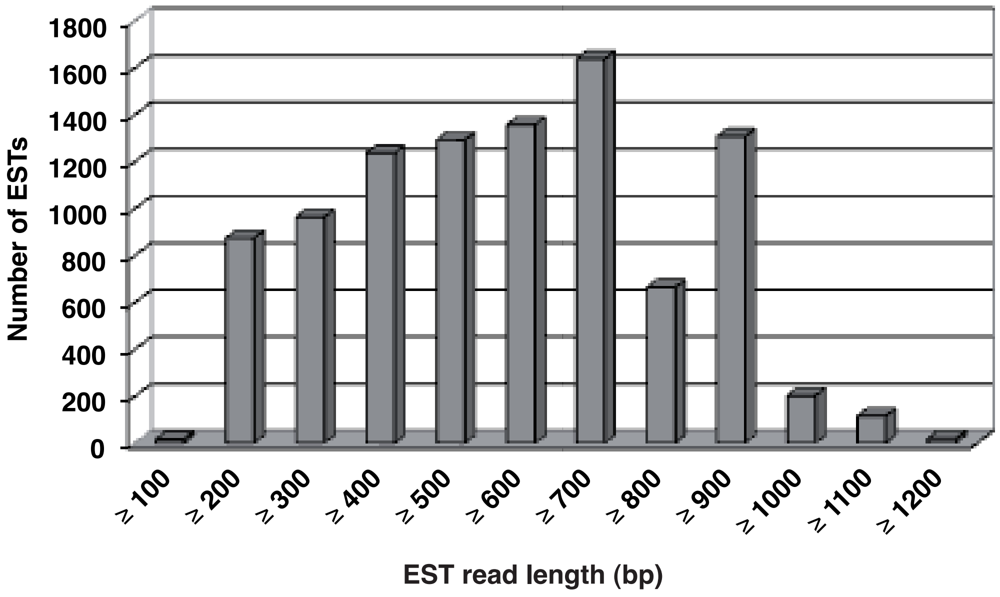

Supplementary Figure 1: EST read length statistics. Read lengths of 9696 ESTs after quality control. The average EST read lenght was 537 bp, read lengths peaked between 700 and 800 bp.

Borchardt et al., Supplementary Figure 2

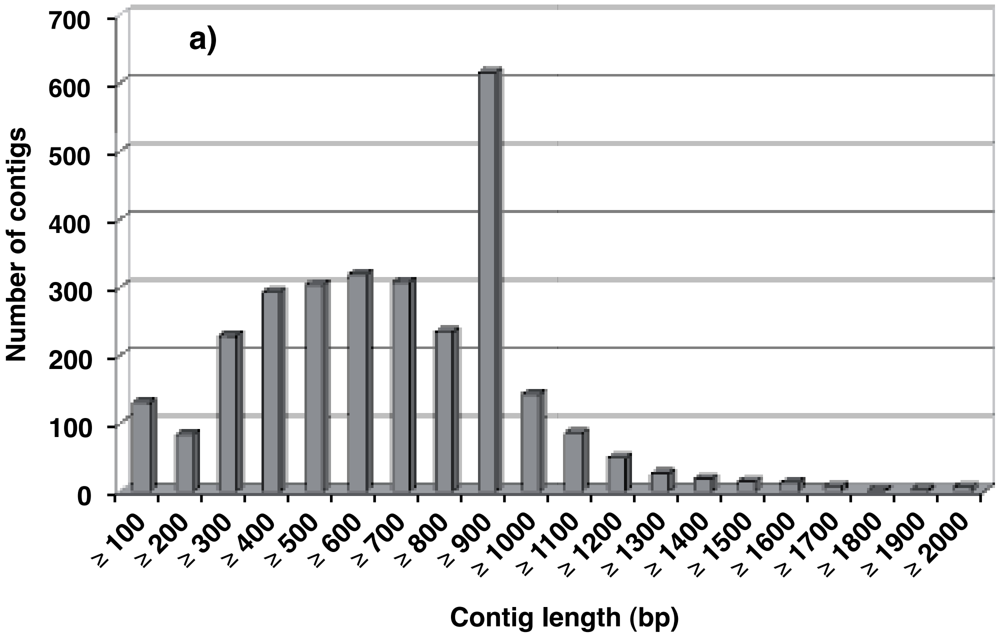

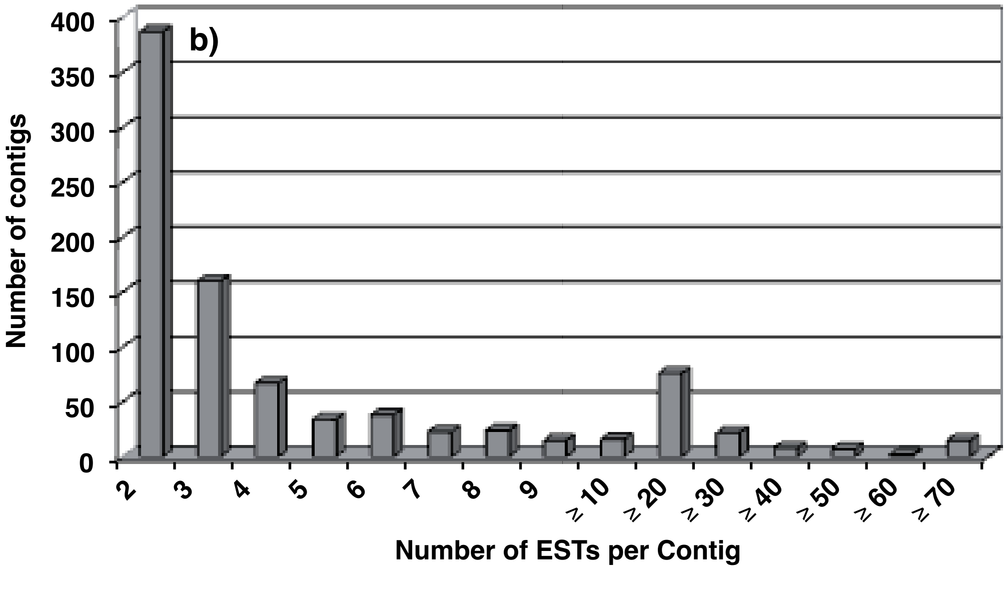

Supplementary Figure 2: Contig length statistics and EST numbers per contig. a) length distribution of 2894 assembled contigs. The average contig length was 627 bp. Peak contig length was between 1000 and 1100 bp. b) number of ESTs per contig containing at least two ESTs. Most of ESTs existed as singletons only, 30,9% of all contaings contained 2 ESTs. The average number of ESTs per contig was 3,1.
