# Supplementary material for: Analysis of newly established EST databases reveals similarities between heart regeneration in newt and fish
Source: BMC Genomics. 2010 Jan 4;11:4. doi: 10.1186/1471-2164-11-4 (PMC2823690; doi:10.1186/1471-2164-11-4)
Supplement: Additional file 4 — Figure S3 shows a graph of GO term nodes used to functionally annotate newt and zebrafish proteins. [file 1471-2164-11-4-S4.PDF]

Borchardt et al., Supplementary Figure 3

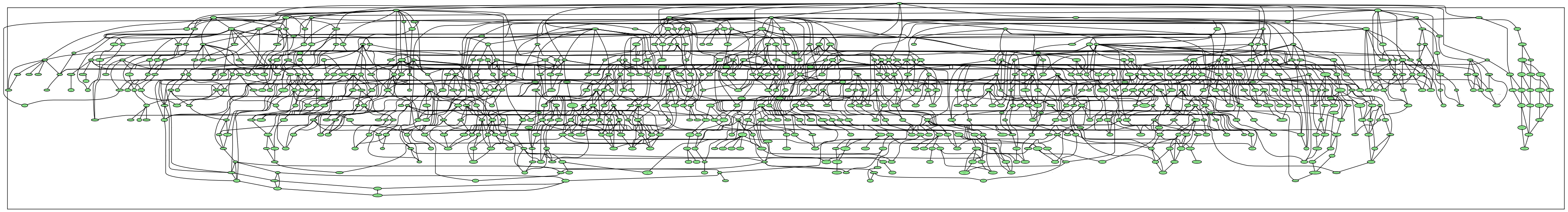

Supplementary Figure 3: GO term tree of all possible term nodes within preselected root nodes donwstream of GO:0008150 biological process. The ancestor terms GO: 0007275: development, GO: 0042060: wound healing, GO: 0016477: cell migration, GO: 0008283 cell proliferation, GO: 0008219: cell death, GO: 0030154: cell differentiation, GO: 0051301: cell division, GO: 0007049: cell cylce, GO: 0000278: mitotic cell cycle, GO: 0008015: circulation, GO: 004007: growth, GO: 0006936: muscle contraction, GO: 0007166: cell surface receptor linked signal transduction, GO: 0030522: intracellular receptor-mediated signaling pathway and GO: 0050851: antigen receptor-mediated signaling pathway were chosen as input GO terms. All possible GO terms downstream in hierarchy were plotted with Blast 2GO.
