# Supplementary material for: Analysis of newly established EST databases reveals similarities between heart regeneration in newt and fish
Source: BMC Genomics. 2010 Jan 4;11:4. doi: 10.1186/1471-2164-11-4 (PMC2823690; doi:10.1186/1471-2164-11-4)
Supplement: Additional file 8 — Figure. Figure S4 demonstrates the features of the newt database. [file 1471-2164-11-4-S8.PDF]

Borchardt et al., Supplementary Figure 4

a)

contig ID: 1 **contig length:** 691 **contig name:** Name folgt

contig sequence:

```
  1 GGAATGACYGCTGTACTTTTACGGAAGACTTCAGTGGCATCCCACGACAACCACCATGACATTCACAAATGATGAGAGAA
 81 AGCACATCCATGACGTCTTGTGCAAAATTGATGCTGGTCATGTAGGAGCCGAATCTCTTGGCAGACTTTTCAATGTCTGC
161 CCATGGACAAGGAGATATTTCAAATCATTTGGTGACCTCAGTACCTGCGAAGCAATCCAGCACAACCCCAAAGTTGCTGC
241 ACACGGTGCCAAGGTGCTACATTCCATTTTGGAGGCGGCCAAACACCTGGATGACCTGAGGGCATACTATGCAGATCTTA
321 GTGTCGTTCACTGCAAAAAACTTTTTGTGGATCCTGCCAACTTCAAGCTGTTTTGTGGAATCGTTAGCATTGTCATCGGC
401 CAGCACCTGGGGCATGACTACACACCTCTGATGCAGTCTGCCTGTGAGAAGTTCCTGCATCACGTTGAGACCGCCCTGGC
481 CTCCGGGTACCACTGATCTATGAAGAAGCATCTCTCTAAGGGTCCTGAAGCTACAAAATGTCCACCGTGGGATACCTTCA
561 TCAACGTACATGAATCAAATATTTTGTGTTAATATGTAGTTGCACTTGTAGCAGCAACACCGAGCAACTAATAAAACAAG
641 CATTGGCAAAGaCAAAAAAAAAAAAAAAAAAAAAAAAAAAAAAAAAAAAAMM
```

Show BLAST hits of the Contig here

number of sequences in conig: 220

Sequences belonging to contig:

sequence ID	sequence incl. vector	sequence length	coordinate (96er)	plate number
1702 Details	GGGGCAAGCGTCTGTAACGACTCTATAGGGCGCTAGCTCGCCGCAGCCGTAACTGACGCGAGCGCAGCGAGTCAGTGAGC...	1088	B_D09	224
601 Details	TTTTTTTATTACCGACTACTATAGGGCGCTAGCTCGCCGCAGCCGAACGACCGAGCGCAGCGAGTCAGTGAGCGAGGAAG...	1288	B_H09	221
10820 Details	ACTGTACGACTACTATAGGGCGCTAGCTCGCCGCAGCCGAACGACCGAGCGCAGCGAGTCAGTGAGCGAGGAAGCGGCCG...	1233	A_F07	248
10122 Details	CTTGGTAAACGACTACTATAGGGCGCTAGCTCGCCGCAGCCGAACGACCGAGCGCAGCGAGTCAGTGAGCGAGGAAGCGG...	1278	B_D03	246
7876 Details	GTTTGTTAACCGACCTTCTATAGGGCGCTAGCTCGCCGCAGCCGAACGACCGAGCGCAGCGAGTCAGTGAGCGAGGAAGC...	1265	B_H07	240
2022 Details	GAGGACTGGATAGTAACGACTACTATAGGGCGCTAGCTCGCCGCACAGGAAGGGCGAGCGAGCGAGTCAGTGAGCGAGGA...	1276	A_G06	225
5995 Details	GAATTCTTGTTAACCGACTACTATAGGGCGCTAGCTCGCCGCAGCCGAACGACCGAGCGCAGCGAGTCAGTGAGCGAGGA...	1312	C_C05	235
7486 Details	CTGTTTACGACTACTATAGGGCGCTAGCTCGCCGCAGCCGAACGACCGAGCGCAGCGAGTCAGTGAGCGAGGAAGCGGCC...	1301	B_G12	239

b)

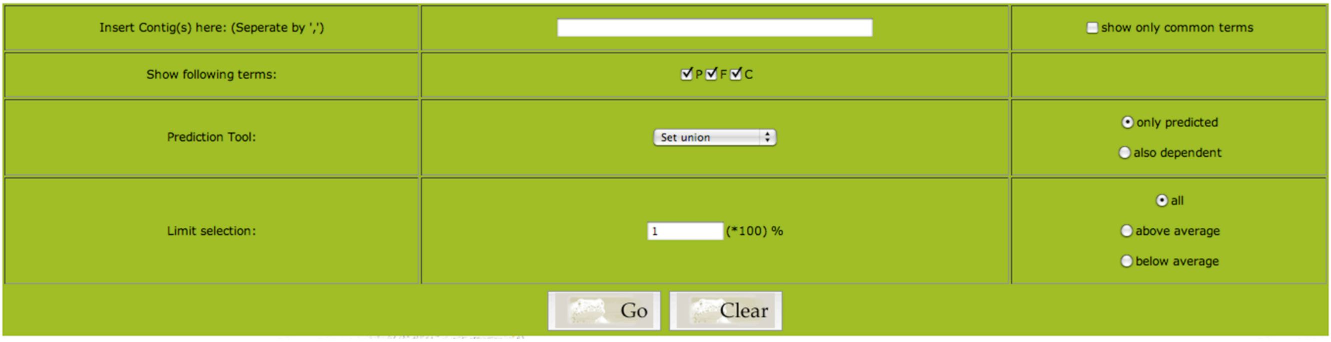

Borchardt et al., Supplementary figure 4

c)

Actual term: GO:0006084, type: P
name: acetyl-CoA metabolism

Specialisation	Actual node	Generalisation
GO:0006085 is_a<br>(D) (T2C) (Tree) (->GOA)	GO:0006084<br>(D) (T2C) (Tree) (->GOA)	is_a GO:0006732<br>(D) (T2C) (Tree) (->GOA)
GO:0019287 is_a<br>(D) (T2C) (Tree) (->GOA)	GO:0006084<br>(D) (T2C) (Tree) (->GOA)	
GO:0019442 is_a<br>(D) (T2C) (Tree) (->GOA)	GO:0006084<br>(D) (T2C) (Tree) (->GOA)	
GO:0019474 is_a<br>(D) (T2C) (Tree) (->GOA)	GO:0006084<br>(D) (T2C) (Tree) (->GOA)	
GO:0046356 is_a<br>(D) (T2C) (Tree) (->GOA)	GO:0006084<br>(D) (T2C) (Tree) (->GOA)	

d)

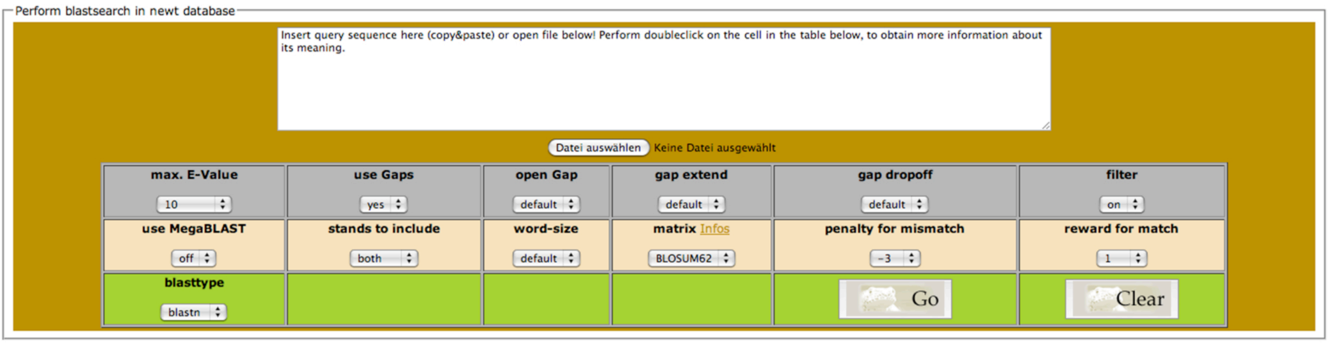

Supplementary figure 4: Features of the Newt EST database. A) Section displaying contig ID, sequence length, consensus DNA sequence, links to BLAST searches for the corresponding contig, the number of ESTs assembled to the contig, and details of plate coordinates and EST sequences. B) GO term viewer, allowing assignment of contigs to GO terms and assignment of GO terms to contigs. C) Display of a selected GO term node including all connected upstream and donwnstream nodes. Links to detailed descriptions of GO terms (D), all contigs assigned to the corresponding GO term (T2C), link to Quick GO (Tree) and links to all proteins deposited in the GOA database assigned to the corresponding GO term (>GOA). D) BLAST search form allowing blastn and tblastn searches of new sequences of the newt EST database with either default or customized parameters.
